# A Deep Learning-Based Ensemble System for Brent and WTI Crude Oil Price Analysis and Prediction

**DOI:** 10.3390/e27111122

**Published:** 2025-10-31

**Authors:** Yiwen Zhang, Salim Lahmiri

**Affiliations:** Department of Supply Chain and Business Technology Management, John Molson School of Business, Concordia University, Montreal, QC H3H 0A1, Canada; yiwen.zhang@mail.concordia.ca

**Keywords:** deep learning, ensemble system, Bayesian optimization, sequential least squares programming, crude oil market, forecasting

## Abstract

Crude oil price forecasting is an important task in energy management and storage. In this regard, deep learning has been applied in the literature to generate accurate forecasts. The main purpose of this study is to design an ensemble prediction system based on various deep learning systems. Specifically, in the first stage of our proposed ensemble system, convolutional neural networks (CNNs), long short-term memory networks (LSTMs), bidirectional LSTM (BiLSTM), gated recurrent units (GRUs), bidirectional GRU (BiGRU), and deep feedforward neural networks (DFFNNs) are used as individual predictive systems to predict crude oil prices. Their respective parameters are fine-tuned by Bayesian optimization (BO). In the second stage, forecasts from the previous stage are all weighted by using the sequential least squares programming (SLSQP) algorithm. The standard tree-based ensemble models, namely, extreme gradient boosting (XGBoost) and random forest (RT), are implemented as baseline models. The main findings can be summarized as follows. First, the proposed ensemble system outperforms the individual CNN, LSTM, BiLSTM, GRU, BiGRU, and DFFNN. Second, it outperforms the standard XGBoost and RT models. Governments and policymakers can use these models to design more effective energy policies and better manage supply in fluctuating markets. For investors, improved predictions of price trends present opportunities for strategic investments, reducing risk while maximizing returns in the energy market.

## 1. Introduction

Due to the continuous growth of the global economy and the acceleration of industrialization, the international energy market, particularly fossil fuels, experiences significant volatility in demand and prices [1]. Key components of global energy consumption, such as crude oil, have price fluctuations that directly impact the global economic structure and influence national policymaking. Increases in energy prices directly impact economic productivity, unemployment, inflation, and the balance of payment equilibrium [2]. Moreover, geopolitical tensions, international trade policies, and currency exchange rate fluctuations further complicate these commodities’ pricing. The complexity of forecasting in this volatile market has been underscored by recent unprecedented events, such as the COVID-19 pandemic, which have compounded these challenges by causing unprecedented shocks to the energy markets, as evidenced by the historic drop on 20 April 2020, when WTI crude oil futures dropped to a negative value at around USD negative 37 per barrel [3]. These dynamics highlight the critical need for more sophisticated forecasting tools to navigate and predict such complex market behaviors effectively.

In recent years, many predictive approaches have been proposed to forecast crude oil prices, reflecting the complex nature of energy markets. They range from traditional statistical approaches to machine learning, deep learning, and hybrid models, illustrating the evolution of forecasting methods in response to the complex dynamics of energy markets. For instance, in [4], the authors utilized traditional statistical techniques such as the autoregressive integrated moving average (ARIMA) process, single exponential smoothing, and k-nearest neighbor to forecast oil, coal, and natural gas prices in India before and after the COVID-19 scenario, highlighting the utility of conventional models in capturing price dynamics. Haque and Shaik [5] concluded that in periods of extreme situations, ARIMA modeling gives good forecasts in predicting WTI. Furthermore, Kristjanpoller and Minutolo [6] introduced a hybrid model that combines artificial neural networks (ANNs) with GARCH to enhance oil price volatility predictions. Their findings demonstrate the potential of merging GARCH’s volatility analysis with ANN’s pattern recognition to tackle market unpredictability.

However, as the dynamic nature of crude oil markets became more apparent, the focus shifted towards integrating machine learning and artificial intelligence techniques to improve forecasting accuracy. For instance, with the advent of computational power, machine learning methods, including support vector machine (SVM) and random forests (RFs), have been extensively applied to price forecasting due to their efficacy in handling non-linear data. For instance, Abdollahi [7] introduced an improved model combining particle swarm optimization for parameter tuning and SVM for forecasting daily crude oil spot prices, showcasing the effectiveness of machine learning techniques. Gupta et al. [8] explored the predictive role of oil-price uncertainty in the UK’s unemployment rate using RF, highlighting the application of machine learning in macroeconomic forecasts.

In addition, deep learning allows the processing of large datasets and the learning of complex non-linear patterns. For instance, long short-term memory (LSTM) and gated recurrent unit (GRU) networks are highlighted for their effectiveness in time series forecasting. Exploring deep learning techniques, particularly LSTM networks, marks a significant advancement in forecasting crude oil prices. Unlike traditional statistical models such as ARIMA and GARCH, which have been staples in analyzing time series data for decades, deep learning approaches offer a nuanced understanding of non-linear relationships and temporal dependencies within the data. Recent studies have demonstrated the application and efficacy of LSTM and GRU models in crude oil price forecasting. For instance, the work by Busari and Lim [9], which integrated AdaBoost with LSTM and GRU models, showcases the potential of deep learning techniques to enhance predictive accuracy in the volatile domain of crude oil prices. Similarly, the innovative study by Huang and Deng [10] employed variational mode decomposition (VMD) in conjunction with LSTM networks, illustrating how combining advanced signal processing with deep learning can yield superior forecasting performance. The study by Lahmiri [11] explores various machine learning systems for forecasting fossil energy market prices. By optimizing these systems with Bayesian method, the research evaluates the Gaussian regression process, support vector regression, regression trees, k-nearest neighbor algorithm, and deep feedforward neural networks. The findings highlight the prediction accuracy of Gaussian regression and prediction stability of deep learning.

In addition, hybrid and ensemble methods combine multiple forecasting models and techniques to overcome the limitations of single models, aiming to improve prediction accuracy and robustness in crude oil forecasting. For example, Li et al. [12] developed hybrid forecasting models that combine VMD with artificial intelligence techniques for monthly crude oil spot price forecasting, including support vector machine and back propagation neural network, both optimized by the genetic algorithm. Ding et al. [13] employed an ensemble model that integrated RF, XG-Boost, and LightGBM to outperform standard single models by improving prediction accuracy.

The main purpose of the current study is to design an ensemble model for crude oil price forecasting. Specifically, the work focuses on deep learning and ensemble methods rather than traditional models like ARIMA and linear regression. This choice stems from the unique characteristics of energy price data. Oil prices show complex, non-linear patterns with sudden changes and sharp fluctuations that simple linear models cannot capture effectively. Moreover, energy prices are non-stationary data, meaning their statistical properties like mean and variance change over time. Traditional time series models like ARIMA are designed for stationary data, where these properties remain constant. Although ARIMA can deal with some level of non-stationarity, it often falls short of capturing the complex and volatile patterns of energy prices [14]. In contrast, deep learning models can automatically learn and adapt to non-linear patterns. Models like LSTM and GRU are especially good at handling time-based data with changing patterns. Similarly, ensemble methods like XGBoost and random forest can pick up complex relationships through their tree structures and boosting techniques.

Building upon these merits of advanced models, the primary goal of this research is to develop a robust, weighted ensemble model that integrates predictions from various deep learning models. While LSTM, GRU, and CNN models are well documented in the literature, there is a potential research gap in systematically comparing and combining these and other deep learning architectures within an ensemble model. This research could fill this gap by identifying which architectures are most effective for different aspects of energy price forecasting.

This ensemble approach aims to address the shortcomings of single-model techniques by combining the strengths of multiple architecture, including CNN, LSTM, BiLSTMs, GRU, BiGRU, and deep feedforward neural networks (DFFNNs). In the first stage of our proposed ensemble system, CNN, LSTM, BiLSTM, GRU, BiGRU, and DFFNN are used as individual predictive systems to predict crude oil price. Their respective parameters are fine-tuned by Bayesian optimization (BO). Additionally, tree-based ensemble methods, namely, extreme gradient boosting (XGBoost) and random forest (RT) are considered as baseline ensemble models.

Focusing on key international crude oil markets (for instance, Brent and WTI), the research aims to optimize predictive performance using Bayesian hyperparameter optimization (BO). This technique will fine-tune the ensemble model by improving the efficiency of hyperparameter selection, ultimately boosting forecast accuracy. Indeed, several studies have underscored the complexity and the risk of overfitting associated with advanced modeling techniques. There is a research opportunity to explore how ensemble methods, combined with BO, might alleviate these concerns. For instance, BO reduces overfitting and simplifies model management by optimizing model parameters and selectively incorporating the most effective models into the ensemble. Levesque et al. [15] noted that when enhanced by BO, ensemble methods adeptly tackle issues like overfitting and model complexity. This is achieved by refining model parameters and strategically selecting optimal models for the ensemble.

To summarize, the main contributions of this research are as follows:

(a) We propose a deep learning-based ensemble model for crude oil market price forecasting; a sequential least squares programming algorithm is employed to aggregate the forecasts.
(b) We apply a diverse set of deep learning models (CNN, LSTM, BiLSTM, GRU, BiGRU, DNN) and standard ensemble methods (XGBoost, RT) for comparison purposes. This is designed to identify market-specific model performance.
(c) We employ BO to fine-tune model parameters to reduce prediction errors and enhance model performance across individual and ensemble models.
(d) We test all models on two major crude oil markets: Brent and WTI.
(e) We provide a detailed comparison between ensemble models and standalone deep learning models, based on various performance metrics.
(f) We utilize a robust dataset spanning from 2010 to 2024, covering Brent and WTI markets. This ensures that all models are trained and validated on real-world data, thereby enhancing their practical applicability.
(g) We seek to develop a forecasting tool that provides actionable insights for policymakers, investors, and energy companies, aiding in better risk management and decision-making processes.

The main findings can be summarized as follows. First, the proposed weighted ensemble system outperforms the individual CNN, LSTM, BiLSTM, GRU, BiGRU, and DFFNN. Second, it outperforms the standard XGBoost and RT models. Third, BiLSTM and BiGRU require optimization to reach better accuracy. Fourth, the BO algorithm improves the accuracy of the weighted ensemble system and GRU. Fifth, the DFFNN performs the least well before and after BO. Fifth, it is concluded that BO improves the accuracy of the predictive models. Sixth, the proposed weighted ensemble model is a promising forecasting tool to predict Brent and WTI daily prices.

The rest of the paper continues as follows: Section 2 describes the predictive models, and Section 3 presents data and provides experimental results. In Section 4, we discuss the work and present our conclusions.

## 2. Materials and Methods

### 2.1. CNN

The convolutional neural network [16] utilizes convolutional layers with learnable filters to capture temporal features automatically in the data without the need for manual feature engineering [17]. Specifically, the core operation within a CNN involves the convolution of filters across the time dimension of the data:

(1) $$f\left(x\right)=\mathrm{ReLU}\left(w\;*\;x+b\right)$$

Here, x is the input sequence, w represents the weights of the convolutional filters, b is the bias, and ∗ denotes the convolution operation. The output is passed through a rectified linear unit (ReLu) activation function to introduce non-linearity, enhancing the model’s ability to learn complex patterns. In this regard, the convolutional layers use filters that slide over the input data to extract local patterns and features, capturing relationships across the temporal dimension. After convolution, an activation function is applied to produce the initial output. This output is then processed by pooling layers, which downsample the feature maps by selecting the maximum or average values within pooling windows, thus reducing the dimensionality while retaining important information. Next, the flattened output from the convolutional and pooling layers forms a one-dimensional vector, which is fed into fully connected (dense) layers. These layers learn high-level representations of the features for classification or regression tasks.

### 2.2. LSTM and BiLSTM

Long short-term memory networks (LSTMs) [18] can capture long-term dependencies within sequential data. The LSTM contains several cells. The cell state mechanism is at the core of its design, composed of a symphony of gates: the input gate $\;(i_{t})$, the forget gate $\left(f_{t}\right)$, and the output gate $\left(o_{t}\right)$, which together manage the flow of information. Weights and biases (W and b) correspond to the weight matrices and bias parameters within the network. The forget gate is given by

(2) $$f_{t}=\sigma \left(W_{f}\left[h_{t-1},X_{t}\right]+b_{f}\right)$$

Here, the forget gate $\left(f_{t}\right)$ regulates the flow of information from the current input and the previous hidden state $\left(h_{t-1}\right)$ by applying a sigmoid activation function, selectively keeping or discarding previous information, guaranteeing that the cell state $\left(C_{t}\right)$ is a dynamic representation of learned data over time. The following step is to use the input gate $\left(i_{t}\right)$ to manage the patterns of data flowing from the current input and the previous hidden states. The cell state ($C_{t}$) updates with contributions from the input gate, the forget gate, the previous cell state ($C_{t-1}$), and the transformed input using the tanh function.

(3) $$i_{t}=\sigma \left(W_{i}\left[h_{t-1},X_{t}\right]+b_{i}\right)$$

(4) $$C_{t}=f_{t}⊙C_{t-1}+i_{t}⊙\tanh\left(W_{c}\cdot \left[h_{t-1},x_{t}\right]+b_{c}\right)$$

Finally, the output gate ($O_{t}$) determines the necessary information for the activation function of the output value ($h_{t}$).

(5) $$O_{t}=\sigma \left(W_{o}\left[h_{t-1},X_{t}\right]+b_{o}\right)$$

(6) $$h_{t}=O_{t}\tanh\left(C_{t}\right)$$

Enhancing the LSTM’s capability, BiLSTMs employ a forward and backward pass through the data. This approach allows the network to capture information from both past and future contexts. The BiLSTM modifies the LSTM’s structure by introducing two separate hidden states for each time step:

(7) $$\vec{h_{t}}=\vec{\mathrm{LSTM}}\left(x_{t},\vec{h_{t-1}}\right)$$

(8) $$\overset{\leftarrow }{h_{t}}=\overset{\leftarrow }{LSTM}\left(x_{t},\overset{\leftarrow }{h_{t+1}}\right)$$

Subsequently, the model joins these states, $\vec{h_{t}}$ and $\overset{\leftarrow }{h_{t}}$, to provide a comprehensive output that captures data in both directions, improving the model’s predicted accuracy.

### 2.3. GRU and BiGRU

The convolutional neural network [16] utilizes convolutional layers with learnable filters to capture temporal features automatically in the data without the need for manual feature engineering [17]. Specifically, the core operation within CNN involves the convolution of filters across the time dimension of the data. Gated Recurrent Units [19] and their bidirectional counterparts (BiGRUs) are integral components of modern sequence modeling within deep learning frameworks. The GRU layer is a type of recurrent neural network that addresses the vanishing gradient problem and can learn long sequences of data [20]. However, the GRU has fewer parameters than the LSTM, leading to better model accuracy [21]. In LSTM, the memory cell state is maintained separately from the hidden state and is updated through three gates: the input gate, the output gate, and the forget gate. Conversely, GRU simplifies this by using a candidate activation vector (${\tilde{h}}_{t}$) instead of a distinct memory cell state and updates it with two gates: the reset gate ($r_{t}$) and the update gate ($z_{t}$). The reset gate controls how much of the previous hidden state should be forgotten, while the update gate determines the extent to which the candidate activation vector should be incorporated into the new hidden state. The equations are represented as follows:

(9) $$z_{t}=\sigma \left(W_{z}\cdot \left[h_{t-1},x_{t}\right]\right)$$

(10) $$r_{t}=\sigma \left(W_{r}\cdot \left[h_{t-1},x_{t}\right]\right)$$

(11) $${\tilde{h}}_{t}=\tanh\left(W_{h}\cdot \left[r_{t}\cdot h_{t-1},x_{t}\right]\right)$$

(12) $$h_{t}=\left(1-z_{t}\right)\cdot h_{t-1}+z_{t}\cdot {\tilde{h}}_{t}$$

Here, the update gate ($z_{t}$) determines how much the unit’s activation or state should be updated. The associated reset gate ($r_{t}$) controls the influence of the previous state on the current computation, allowing GRUs to dynamically discard or retain information across time steps. The candidate activation vector (*h*${\tilde{h}}_{t}$) is computed from the current input ($x_{t}$) and a modified version of the previous hidden state that is adjusted by the reset gate. The new hidden state ($h_{t}$) is then derived by combining the candidate activation vector with the previous hidden state ($h_{t-1}$), with the contribution of each weighted by the update gate.

In addition, the BiGRU enhances the functionality of GRUs by processing data in both forward and backward directions, encapsulating a broader temporal context. The formulas for the BiGRU model are as follows:

(13) $$\vec{h_{t}}=\vec{\mathrm{GRU}}\left(x_{t},\vec{h_{t-1}}\right)$$

(14) $$\overset{\leftarrow }{h_{t}}=\overset{\leftarrow }{GRU}\left(x_{t},\overset{\leftarrow }{h_{t+1}}\right)$$

### 2.4. DFFNN

Deep feedforward neural networks (DFFNNs) represent a widely used type of artificial neural network with multiple hidden layers, designed to model complex functions by increasing network depth [21]. They typically consist of multiple hidden layers, each containing numerous neuron nodes. The architecture ensures that each neuron in one layer is connected to every neuron in the next layer. This structure helps the network learn the best weights for representing the data, which is essential for tasks such as classification or regression. The DFFNN model consists of an input layer, multiple hidden layers (k ≥ 2), and an output layer. The input layer receives the initial data, represented by the input vector x = [$x_{1}$, $x_{2}$,…, $x_{n}$], where n denotes the number of input features. The data is then passed through a series of transformations in the hidden layers. For each hidden layer k, the input undergoes a linear transformation followed by a non-linear activation function. Specifically, the linear transformation involves multiplying the input by a weight matrix $W_{k}$ and adding a bias vector $b_{k}$. This results in the pre-activation value, $z_{k}$. The pre-activation value is then passed through an activation function *f* to produce the activation output $h_{k}$. This process can be summarized by the following equations:

(15) $$z_{k}=W_{k}h_{k-1}+b_{k}$$

(16) $$h_{k}=f\left(z_{k}\right)$$

where $h_{k-1}$ is the output from the previous layer (or the input vector x for the first hidden layer). Finally, the output layer takes the output from the last hidden layer $h_{L-1}$ as its input and applies a similar transformation. The pre-activation value for the output layer is computed as

(17) $$z_{L}=W_{L}h_{L-1}+b_{L}$$

This pre-activation value is then passed through an activation function g to produce the final output y:

(18) $$y=g\left(z_{L}\right)$$

The performance of DFFNNs can be influenced by various hyperparameters, such as the activation function, dropout regularization, and network architecture [22]. This study tests three activation functions in the BO process: rectified linear unit (relu) function, sigmoid function, and hyperbolic tangent (tanh) function.

### 2.5. XGBoost

Extreme gradient boosting is an advanced machine learning technique that improves the performance of decision trees through a boosting framework. It is designed to improve both the speed and accuracy of predictions of the standard gradient boosting decision tree [23]. In this regard, XGBoost builds an ensemble of weak learners through an iterative process. For instance, each new decision tree is trained to correct the errors made by the previous trees, thus improving the model’s accuracy [24]. One of the key features of XGBoost is its regularized objective function, which combines a loss function that measures prediction error with a regularization term to limit model complexity. By controlling the number of leaves in each tree and adjusting the weights of the leaf nodes, XGBoost helps prevent overfitting and improves the model’s ability to generalize [25]. The algorithm starts by making an initial prediction, usually the average of the target variable. It then calculates the errors and constructs decision trees to minimize them. The final prediction is the weighted sum of the predictions from all the trees, as shown in the following equation:

(19) $$\hat{y}=\sum_{k=1}^{K}f_{k}\left(x_{i}\right)$$

where $\hat{y\;}$ is the final prediction, K is the number of trees, and $f_{k}\left(x_{i}\right)$ is the prediction from the k-th tree for input $x_{i}$. To prevent overfitting, XGBoost incorporates a regularization term into the objective function:

(20) $$L\left(\theta \right)=\sum_{i=1}^{n}l\left(y_{i},\hat{y_{i}}\right)+\sum_{k=1}^{K}\Omega \left(f_{k}\right)$$

where $l\left(y_{i},\hat{y_{i}}\right)$ represents the loss function measuring the difference between the actual value $y_{i}$ and predicted value $\hat{y_{i}}$, and $\Omega \left(f_{k}\right)$ is the regularization term controlling the complexity of the k-th tree. The regularization term helps prevent overfitting and is defined as

(21) $$\Omega \left(f_{k}\right)=\gamma T+\frac{1}{2}\lambda \sum_{j=1}^{T}w_{j}^{2}$$

where T is the number of leaf nodes in the tree,$\;w_{j}$ is the weight of the j-th leaf, γ controls the penalty on the number of leaves, and λ is the L2 regularization term that penalizes large leaf weights.

### 2.6. Random Forest

Random forest (RT) [26] is a bagging-based ensemble method that builds multiple decision trees using bootstrap sampling and random feature selection. Hence, this approach reduces overfitting and improves prediction accuracy compared to single decision trees. The algorithm introduces randomness by training each tree on a bootstrap sample of the data and selecting a random subset of features for splitting at each node. For regression problems, the final prediction is the average of all the trees:

(22) $$\hat{y}=\frac{1}{T}\sum_{t=1}^{T}f_{t}\left(x\right)$$

where T is the number of trees, and $f_{t}\left(x\right)$ is the prediction from the t-th tree.

### 2.7. Bayesian Optimization

Bayesian optimization (BO) [27] has emerged as a compelling strategy for hyperparameter tuning in machine learning, proving to be highly beneficial for optimizing complex deep neural networks. BO employs Bayes’ theorem to navigate and optimize the hyperparameter search space systematically. This search space typically includes crucial hyperparameters, such as the number of neurons, learning rates, and network depth, which depend on the structure of machine learning models. At the core of the BO methodology is the application of Bayes’ theorem, which is expressed mathematically as

(23) $$p\left(f|D\right)=\frac{p\left(D|f\right)\cdot p\left(f\right)}{p\left(D\right)}$$

where $p\left(f|D\right)$ represents the posterior probability of the function f after observing the data D, $p\left(D|f\right)$ is the likelihood of observing the data given the function, $p\left(f\right)$ denotes the prior belief about the distribution of function f , and $p\left(D\right)$ serves as the normalizing constant. In this regard, BO employs Bayes’ theorem to refine its model of hyperparameter performance iteratively, integrating data from new evaluations. Hence, the probabilistic updating process enables BO to recalibrate the search space exploration dynamically. Specifically, the probabilistic updating process balances the exploration of unexplored parameter configurations with the exploitation of regions known to produce favorable outcomes. This strategic balance is critical as it directs BO’s computational efforts towards the most promising areas of the search space. The strategic utilization of BO has been demonstrated to significantly reduce the mean square error and enhance the overall performance of models across various settings [28]. In addition, Wang et al. [29] showed how sophisticated optimization techniques like BO can revolutionize the field of machine learning by boosting efficiency and diminishing the resources required for model training.

### 2.8. Proposed Deep Learning-Based Ensemble Model

**Figure 1:**
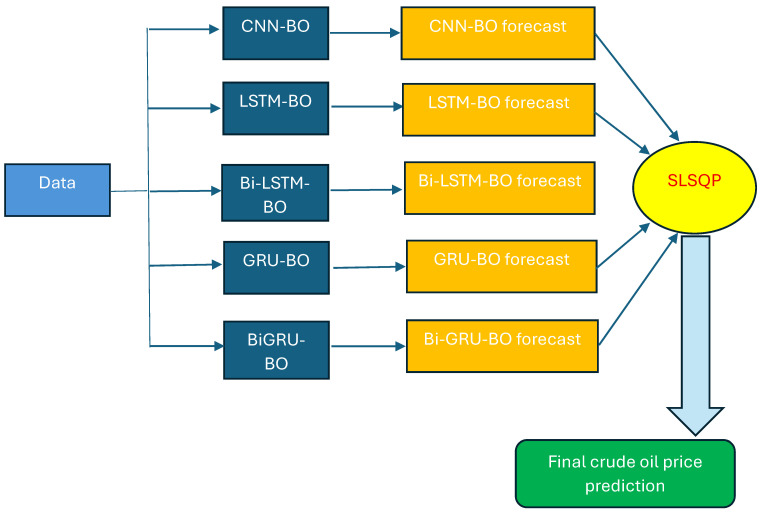
Flowchart of our proposed weighted ensemble based on deep learning systems to predict the next-day spot price of Brent and WTI. In the proposed ensemble of deep learning systems, each single deep learning model is used to generate a forecast in the first stage. In the second stage, the sequential least squares programming (SLSQP) algorithm is employed to compute the final prediction. The Bayesian optimization algorithm is embedded in each model to fine-tune its parameters.

Our proposed mode for crude oil price prediction is based on an ensemble of deep learning systems where each single deep learning is used to generate a forecast in the first stage. Then, in the second stage, the sequential least squares programming (SLSQP) [30] algorithm is adopted to calculate the final prediction. The latter is calculated by SLSQP as the weighted value from all forecasts generated by deep learning systems. Specifically, in this stage, the SLSQP algorithm is used to minimize the mean squared error between the actual values and the weighted sum of the ensemble model predictions. The algorithm adjusts the weights of CNN, LSTM, BiLSTM, GRU, BiGRU, and DFFNN, with the constraint that the weights add up to 1. SLSQP is an iterative method for constrained non-linear optimization with significant potential to be more stable than the sequential quadratic programming algorithm under numerical noise [31]. Figure 1 shows the flowchart of our proposed deep learning-based ensemble model, where BO is used to fine-tune parameters of deep learning systems and SLSQP is adopted for final forecast aggregation. It is expected that our ensemble model based on deep learning systems could benefit from their respective advantages to generate accurate predictions.

### 2.9. Performance Measures

Our proposed method for crude oil price prediction is based on an ensemble of deep learning systems where each single deep learning is used to generate a forecast in the first stage. Then, in the second stage, the sequential least squares programming (SLSQP) [30] algorithm is adopted to calculate the final prediction. The latter is calculated by SLSQP as the weighted value from all forecasts generated by deep learning systems. Specifically, in this stage, the SLSQP algorithm is used to minimize the mean squared error between the actual values and the weighted sum of the ensemble model predictions. The algorithm adjusts the weights of CNN, LSTM, BiLSTM, GRU, BiGRU, and DFFNN, with the constraint that the weights add up to 1. The SLSQP is an iterative method for constrained non-linear optimization with significant potential to be more stable than the sequential quadratic programming algorithm under numerical noise [31]. Figure 1 shows the flowchart of our proposed deep learning-based ensemble model, where BO is used to fine-tune parameters of the deep learning systems and SLSQP is adopted for final forecast aggregation. It is expected that our ensemble model based on deep learning systems could benefit from their respective advantages to generate accurate predictions. In this work, a range of evaluation metrics are used to evaluate the performance of each model, including the root mean squared error (RMSE), mean absolute error (MAE), and mean absolute percentage error (MAPE), as they are the most common metrics employed in time series forecasting [32,33].

## 3. Results

**Figure 2:**
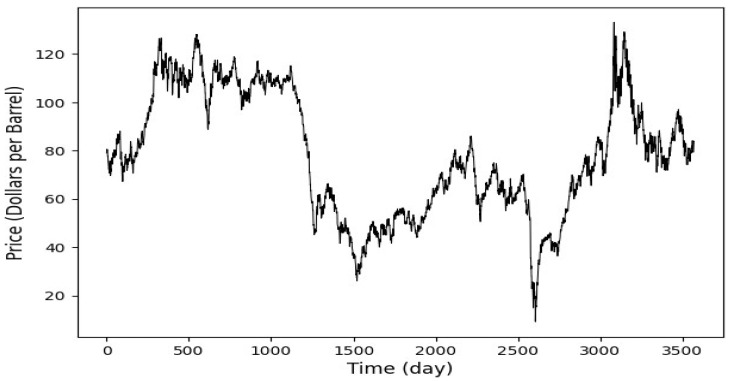
Brent daily spot price. The datasets span from 4 January 2010 to 12 February 2024. The dataset is split into 80% for training the predictive models (4 January 2010, to 9 September 2019) and the remaining 20% (from 10 September 2019 to 12 February 2024) for testing.

**Figure 3:**
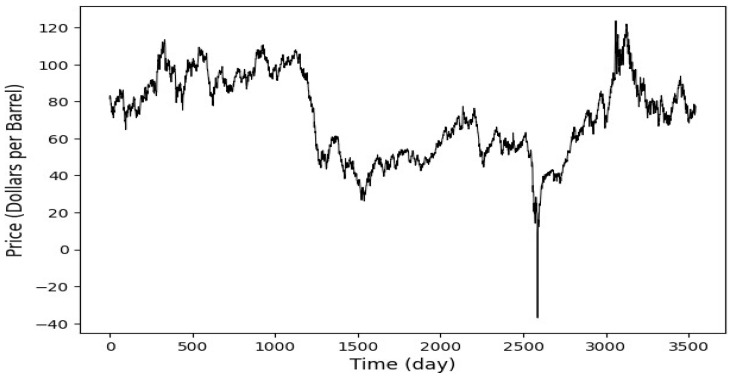
WTI daily spot price. The dataset is split into 80% for training the predictive models (4 January 2010 to 9 September 2019) and the remaining 20% (from 10 September 2019 to 12 February 2024) for testing.

This study implements and proposes an ensemble deep learning models to predict spot prices in two major crude oil markets: Brent and WTI. The datasets span from 4 January 2010, to 12 February 2024, and feature daily observations. The datasets were obtained from the U.S. Energy Information Administration (EIA) [34], all in USD. Collecting over a decade of data enhances the deep learning models used in this research by providing a broad spectrum of market conditions, which helps detect complex patterns and improve forecasts’ accuracy. This extensive data collection enables the models to effectively generalize and predict future market behaviors, which is crucial for achieving robust performance in the volatile markets of oil. Figure 2 and Figure 3, respectively, display Brent and WTI spot prices. In this study, a normalization procedure is applied to scale the data between 0 and 1, ensuring consistency in data scales and facilitating efficient model training. The dataset is split into 80% for training (4 January 2010, to 9 September 2019) and the remaining 20% (from 10 September 2019, to 12 February 2024) for testing, allowing for robust training and performance evaluation on unseen data. A 30-day historical window is used to forecast one day ahead, capturing essential trends in Brent and WTI crude oil markets. Each training instance uses a 30-day sequence to predict the next day’s value, enhancing the model’s ability to learn from recent behaviors. This method aligns with standard financial and commodity market analysis practices and ensures reliable prediction performance. The hyperparameters setting is shown in Table 1.

**Figure 4:**
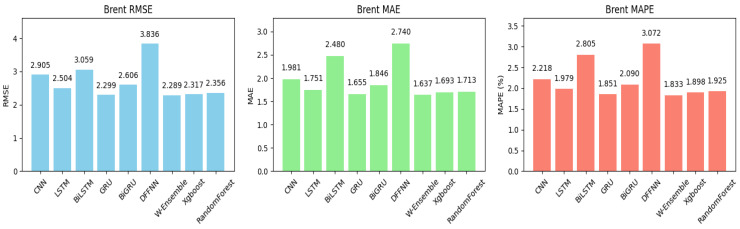
Bar plots of performance metrics across all models post-BO for the Brent crude oil market. Under Bayesian optimization, the proposed weighted ensemble system (W-Ensemble) reached the lowest RMSE, MAE, and MAPE. The DFFNN system obtained the highest RMSE, MAE, and MAPE.

Table 2 presents the forecasting performance of various models for Brent oil using three evaluation metrics, MAE, RMSE, and MAPE, before and after Bayesian Optimization (BO). Overall, most models improved all three metrics after BO, indicating that the optimization enhanced their predictive accuracy. Looking at RMSE, several models experienced notable improvements. The models that showed the most improvement were LSTM, BiLSTM, CNN, GRU, and XGBoost. For example, the RMSE of LSTM dropped from 3.726 to 2.504, a significant reduction of about 32.8%. After BO, our proposed model, the weighted ensemble model, achieved the best performance, with the lowest RMSE of 2.289. This suggests that combining multiple models (ensemble) helps capture complex patterns better than individual models, resulting in lower prediction errors. The next best individual model was GRU, with an RMSE of 2.299. XGBoost performed better than random forest, with an RMSE of 2.317. On the other hand, the worst-performing model post-BO was the DFFNN, with an RMSE of 3.836, indicating that this model struggled to improve despite optimization. Figure 4 exhibits the bar plots for performance of all models post-BO when applied to Brent data for better visualization of the forecasting results.

**Figure 5:**
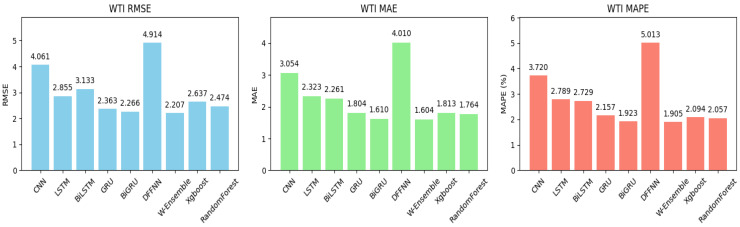
Bar plots of performance metrics across all models post-BO for the WTI crude oil market. Under Bayesian optimization, the proposed weighted ensemble system (W-Ensemble) reached the lowest RMSE, MAE, and MAPE. The DFFNN system obtained the highest RMSE, MAE, and MAPE.

For the WTI crude oil market, as shown in Table 3, before BO, the models exhibited varying performance. Random forest had the lowest RMSE at 2.491, likely due to its ability to capture non-linear patterns through its ensemble structure. GRU also performed well with an RMSE of 2.720, reflecting its strength in handling sequential data. In contrast, more complex models like BiLSTM and BiGRU had higher RMSEs, possibly due to overfitting or the need for further tuning. After applying BO, our proposed model, the weighted ensemble model, once again achieved the best results, with the lowest RMSE of 2.207, confirming the value of ensemble methods in improving accuracy. The DFFNN, however, continued to perform poorly, with an RMSE of 4.914, indicating it is not well-suited to this task without further refinement. Figure 5 exhibits the bar plots for performance of all models post-BO when applied to WTI for better visualization of the forecasting results.

**Figure 6:**
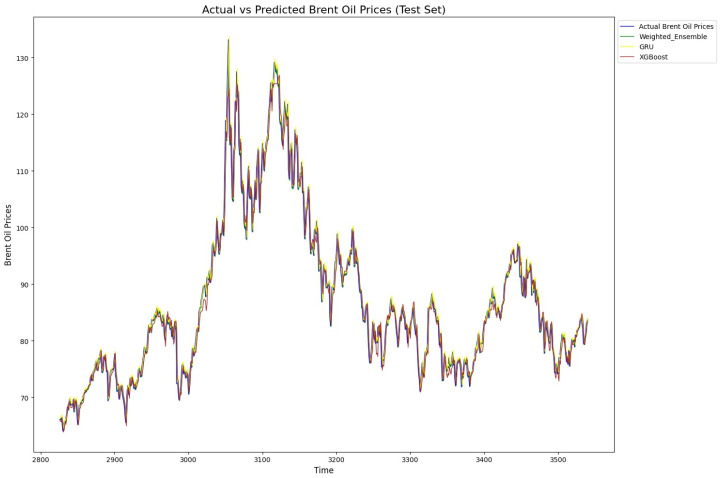
Actual and predicted prices of Brent by proposed weighted model, GRU, and XGBoost. All three models fit the true prices well, showing their ability to model non-linearity, sudden changes, and trends in Brent price time series.

**Figure 7:**
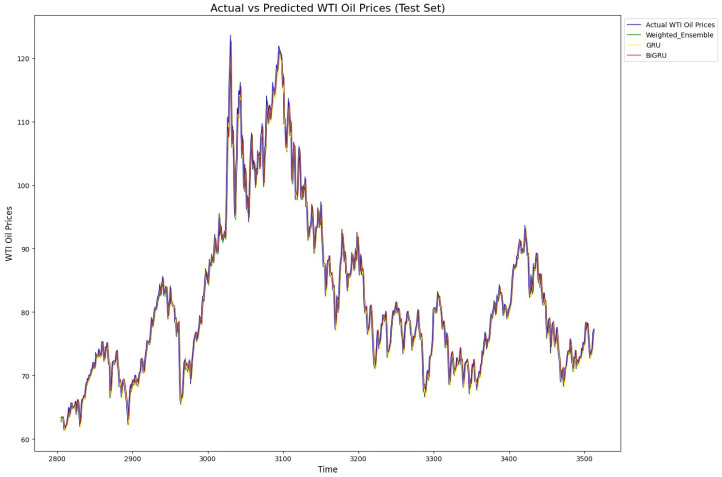
Actual and predicted prices of WTI by proposed weighted model, GRU, and XGBoost. All three models fit the true prices well, showing their ability to model non-linearity, sudden changes, and trends in WTI price time series.

In summary, the analysis shows that simpler models like random forest and GRU were more robust and performed better pre-BO, while more complex models like BiLSTM and BiGRU needed optimization to reach their full potential. After BO, weighted ensemble models and GRU-based models excelled in the oil market predictions, while simpler neural networks like DFFNNs faced difficulties in achieving competitive results. Finally, for illustration, Figure 6 and Figure 7 exhibit the predicted versus true value for our proposed model, GRU, and XBoost when applied to Brent and WTI data, respectively. As shown, the models align well with the actual value patterns, showcasing their impressive predictive capabilities. The models consistently capture the trends and fluctuations in each market, reflecting their robustness and accuracy in handling complex time series data. This alignment with actual values underscores the effectiveness of these models in real-world market prediction.

## 4. Discussion and Conclusions

Time series forecasting is a major task in business and engineering. Indeed, thanks to their superior performance, in recent years, machine learning and deep learning have been widely employed to forecast stock prices [35,36,37,38,39,40,41,42,43,44,45,46,47,48,49], energy commodity prices [50,51,52,53,54,55,56], and macroeconomic indicators [57].

This study tackles the challenge of forecasting prices in the highly volatile oil markets, which play a crucial role in the global economy. Price fluctuations in these markets have significant impacts on national economies, energy policies, and investment decisions. Our proposed model developed in this research aims to enhance forecasting accuracy, offering valuable insights for risk management and informed decision-making as this topic is receiving growing attention in the literature [35,36]. For both Brent and WTI markets, our proposed weighted ensemble model emerged as the top performer. This model excels in capturing both short- and long-term patterns in the data and effectively managing the volatility inherent in crude oil prices. As a result, our proposed model is a strong option for decision-makers looking to minimize forecasting errors in this sector.

Conversely, the worst-performing model in both oil and gas markets was the standalone DFFNN. While it can capture general trends in the original data, it struggles with the sequential and time-dependent nature of energy prices. DFFNNs are more difficult to train because of issues like overloading hidden units and vanishing or exploding gradients [37]. This limitation makes them less effective in volatile markets like crude oil markets, where capturing dependencies over time is crucial. In addition, Bayesian Optimization played a crucial role in enhancing the performance of deep learning models and ensemble models by systematically optimizing their respective hyperparameters by using a probabilistic approach to minimize errors effectively.

Indeed, in this study, BO led to significant improvements in all models’ performance, especially for BiLSTM, GRU, Bi- GRU, and XGBoost. This research presents a direct comparison between deep learning models (such as CNN, LSTM, BiLSTM, GRU, and BiGRU) and standard ensemble models (like XGBoost and Random Forest), an area that has been less thoroughly explored in the current literature. While earlier studies often focused on either deep learning or ensemble models in isolation, our findings demonstrate that ensemble methods, particularly when fine-tuned using Bayesian Optimization (BO), can match or even exceed the performance of deep learning techniques. In summary, this research significantly contributes to the field by demonstrating the effectiveness of using advanced models and optimization techniques for forecasting energy prices, namely, crude oil markets.

Overall, our proposed weighted ensemble based on deep learning systems and optimized through BO offers practical solutions for managing the inherent risks and uncertainties of crude oil markets, providing stakeholders with more reliable forecasts for informed decision-making. For instance, more accurate forecasting models enable managers and traders to improve pricing strategies, inventory management, and hedging tactics, ultimately resulting in more profitable and data-driven decision-making. Governments and policymakers can use these models to design more effective energy policies and better manage supply in fluctuating markets. For investors, improved predictions of price trends present opportunities for strategic investments, reducing risk while maximizing returns in the energy market. In summary, the proposed weighted ensemble based on deep learning systems and optimized through BO is recommended to investors and traders to produce accurate forecasts to better generate profits and control risk.

While this research has made significant progress in forecasting fossil energy markets, there is a limitation. The models were tested only on crude markets, leaving other energy markets like coal, gasoline, or propane unexplored. Since different energy markets may show different price patterns, as a result, the performance of certain models could vary, and other models may perform better in these markets.

For future work, the forecasting of spot prices of other fossil energy markets will be considered. In addition, the model will be applied to predict volatility in crude oil and other fossil energy markets. Furthermore, another avenue of applications of the proposed ensemble deep learning models comprises clean energy markets, including the forecasting of spot prices and volatilities. Indeed, such investigations would help verify the effectiveness of the proposed ensemble deep learning systems across different energy markets.

## Figures and Tables

**Table 1:** Hyperparameter settings.

Model	Parameter	Value Range and Setup
Conv1D	Filters	(16, 256, 16)
	Kernel size	[3, 5]
	Dense units	(8, 128, 8)
	Learning rate	(0.0001, 0.01, Log-uniform)
Uni/BiLSTM	Units	(16, 256, 16)
	Learning rate	(0.0001, 0.01, Log-uniform)
Uni/BiGRU	Units	(16, 256, 16)
	Learning rate	(0.0001, 0.01, Log-uniform)
DFFNN	Hidden layers	(1, 5)
	Network neurons	(10, 100)
	Activation	(0, 2)
XGBoost	Max depth	(3, 10)
	Learning rate	(0.01, 0.3, Log-uniform)
	N estimators	(100, 1000)
	Min child weight	(1, 10)
	Subsample	(0.5, 1.0, Uniform)
	Colsample bytree	(0.5, 1.0, Uniform)
Random forest	Max depth	(3, 20)
	N estimators	(100, 1000)
	Min samples split	(2, 10)
	Min samples leaf	(1, 4)
	Max features	[‘sqrt’, ‘log2’, None]
	Bootstrap	[True, False]

Note: activation (0, 2)—index for activation functions; maps to relu, sigmoid, tanh.

**Table 2:** Forecasting performance on the testing set of all models for Brent before and after applying Bayesian optimization to tune the hyperparameters of the predictive models. Under Bayesian optimization, the proposed weighted ensemble deep learning model outperforms all reference models. Bayesian optimization improves the performance of all models, except the DFFNN system. Bold indicates the best performance without and with optimization.

Models	Pre-BO			Post-BO		
	RMSE	MAE	MAPE	RMSE	MAE	MAPE
**1D-CNN**	3.754	2.720	3.050%	2.905	1.981	2.218%
**LSTM**	3.726	2.666	3.010%	2.504	1.751	1.979%
**BiLSTM**	4.523	3.250	3.690%	3.059	2.480	2.805%
**GRU**	2.876	2.046	2.290%	2.299	1.655	1.851%
**BiGRU**	2.768	1.977	2.210%	2.606	1.846	2.090%
**DFFNN**	3.292	2.297	2.570%	3.836	2.740	3.072%
**XGBoost**	2.736	1.997	2.230%	2.317	1.693	1.898%
**Random forest**	**2.377**	**1.737**	**1.950%**	2.356	1.713	1.925%
**Our model: weighted ensemble deep learning**	3.276	2.314	2.600%	**2.289**	**1.637**	**1.833%**

**Table 3:** Forecasting performance on testing set of all models for WTI before and after applying Bayesian optimization to tune the hyperparameters of the predictive models. Under Bayesian optimization, the proposed weighted ensemble deep learning model outperforms all reference models. Bayesian optimization improves the performance of all models, except the 1D-CNN, DFFNN, and random forest system. Bold indicates the best performance without and with optimization.

Models	Pre-BO			Post-BO		
	RMSE	MAE	MAPE	RMSE	MAE	MAPE
**1D-CNN**	3.477	2.525	3.010%	4.061	3.054	3.720%
**LSTM**	4.139	3.105	3.680%	2.855	2.323	2.789%
**BiLSTM**	3.778	2.725	3.270%	3.133	2.261	2.729%
**GRU**	2.720	2.053	2.430%	2.363	1.804	2.157%
**BiGRU**	3.348	2.608	3.130%	2.266	1.610	1.923%
**DFFNN**	3.508	2.658	3.190%	4.914	4.010	5.013%
**XGBoost**	3.112	2.118	2.440%	2.637	1.813	2.094%
**Random forest**	**2.491**	**1.796**	**2.100%**	2.474	1.764	2.057%
**Our model: weighted ensemble deep learning**	3.307	2.452	2.930%	**2.207**	**1.604**	**1.905%**

## Data Availability

Data source is described in the paper.

## Abbreviations

The following abbreviations are used in this manuscript:

CNN	Convolutional neural network
LSTM	Long short-term memory
BiLSTM	Bidirectional LSTM
GRU	Gated recurrent unit
BiGRU	Bidirectional GRU
DFFNN	Deep feedforward neural network
BO	Bayesian optimization
SLSQP	Sequential least squares programming
XGBoost	Extreme gradient boosting
RT	Random forest
WTI	West Texas Intermediate
RMSE	Root mean of squared error
MAE	Mean absolute error
MAPE	Mean absolute percentage error
